# Establishing the functional relevancy of white matter connections in the visual system and beyond

**DOI:** 10.1007/s00429-021-02423-4

**Published:** 2021-11-30

**Authors:** Mareike Grotheer, Emily Kubota, Kalanit Grill-Spector

**Affiliations:** 1 Department of Psychology, Philipps-Universität Marburg, 35032 Marburg, Germany; 2 Center for Mind, Brain and Behavior-CMBB, Philipps-Universität Marburg and Justus-Liebig-Universität Giessen, 35037 Marburg, Germany; 3 Psychology Department, Stanford University, Stanford, CA 94305 USA; 4 Wu Tsai Neurosciences Institute, Stanford University, Stanford, CA 94305 USA

**Keywords:** White matter, Fascicles, Functional magnetic resonance imaging, Diffusion magnetic resonance imaging, Precision neuroscience

## Abstract

For over a century, researchers have examined the functional relevancy of white matter bundles. Consequently, many large-scale bundles spanning several centimeters have been associated in their entirety with specific brain functions, such as language or attention. However, these coarse structural–functional relationships are at odds with modern understanding of the fine-grained functional organization of human cortex, such as the mosaic of category-selective regions in ventral temporal cortex. Here, we review a multimodal approach that combines fMRI to define functional regions of interest within individual’s brains with dMRI tractography to identify the white matter bundles of the same individual. Combining these data allows to determine which subsets of streamlines within a white matter bundle connect to specific functional regions in each individual. That is, this approach identifies the functionally defined white matter sub-bundles of the brain. We argue that this approach not only enhances the accuracy of interpreting the functional relevancy of white matter bundles, but also enables segmentation of these large-scale bundles into meaningful functional units, which can then be linked to behavior with enhanced precision. Importantly, this approach has the potential for making new discoveries of the fine-grained functional relevancy of white matter connections in the visual system and the brain more broadly, akin to the flurry of research that has identified functional regions in cortex.

## White matter bundles of the human brain

The white matter of the human brain contains a complex architecture of structural connections of different length and size that connect brain regions and enable them to communicate with each other. Large-scale connections that link distant parts of the brain are referred to as white matter fascicles. Most fascicles were initially identified in ex vivo dissection studies and later modeled from in vivo diffusion magnetic resonance imaging (dMRI) data, whereas anatomical priors from the dissection literature are used to guide dMRI analyses. dMRI tractography uses the diffusivity of water molecules within each voxel to estimate the trajectory of white matter connections, as water moves more freely along a connection than perpendicular to it (for a recent review, see Jeurissen et al. (2019)). Nonetheless, dMRI tractography is not without limitations (e.g. Bastiani et al. 2012; Jones et al. 2013; Thomas et al. 2014; Reveley et al. 2015; Maier-Hein et al. 2017) and can only provide a model of the fascicles. As such, here, we will use the term “bundle” when we refer to white matter fascicles estimated from dMRI data. Each bundle is made up of a large number of streamlines, i.e., individual lines traced in dMRI tractography. Importantly, we also use the term sub-bundle, which we define as a subset of streamlines within a bundle that form a meaningful functional and/or structural unit.

**Fig. 1:**
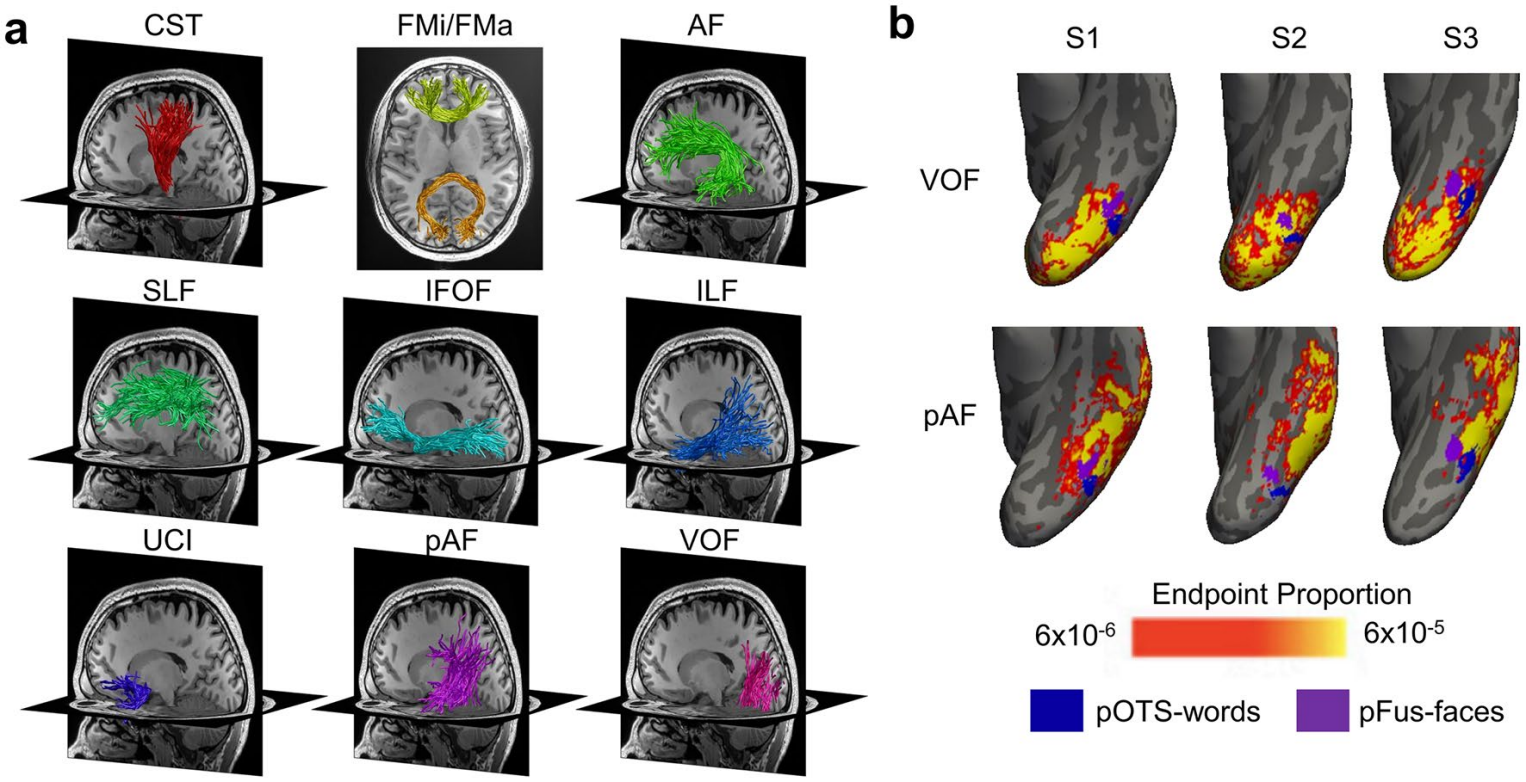
White matter bundles connect distant regions in the brain. **a** Examples of commonly investigated fascicles modeled with dMRI tractography. All panels shows bundles in the same representative individual. *CST* cortico-spinal tract, *FMa* forceps major, *FMi* forceps minor, *AF* arcuate fasciculus, *pAF* posterior arcuate fasciculus, *SLF* superior longitudinal fasciculus, *IFOF* inferior fronto-occipital fasciculus, *ILF* inferior longitudinal fasciculus, *UCI* uncinate fasciculus, *VOF* vertical occipital fasciculus. **b** The proportion of VOF and pAF endpoints (yellow-reds) relative to category-selective regions (word-selective pOTS-words (blue), and face-selective pFus-faces (purple)) in the ventral temporal cortex. Probabilistic endpoint maps and fROIs are shown on the left inflated cortical surfaces of three example individuals. Endpoints of these bundles span a much larger spatial expanse than the fROIs and consequently connect to multiple functional regions. For example, the VOF endpoints connect to both face- and word-selective regions. *pOTS:* posterior occipito-temporal sulcus, *pFus*: posterior fusiform gyrus, *fROI*: functional region of interest

Fascicles of the brain are often divided into three main types: (1) projection fascicles, which connect between sub-cortical and cortical regions (e.g., the cortico-spinal tract (CST)), (2) commissural fascicles, which connect between the two hemispheres (e.g., forceps major (FMa) and forceps minor (FMi)), and (3) association fascicles, which connect distant cortical regions within a hemisphere. Well-studied association fascicles include: (1) the arcuate fasciculus (AF), which connects the frontal and temporal cortices (Catani et al. 2002), (2) the superior longitudinal fasciculus (SLF), which connects the frontal and parietal cortices (Catani et al. 2002), (3) the inferior fronto-occipital fasciculus (IFOF), which connects the frontal and occipital cortices (Catani et al. 2002), (4) the inferior longitudinal fasciculus (ILF), which connects the occipital cortex to the tip of the temporal lope (Catani et al. 2002), (5) the uncinate fasciculus (UCI), which connects the tip of the temporal lobe with the frontal cortex (Catani et al. 2002), (6) the vertical occipital fasciculus (VOF), which connects the ventral and dorsal occipital cortices (Yeatman et al. 2014; Takemura et al. 2016b; Weiner et al. 2016; Bullock et al. 2021) and (7) the posterior arcuate fasciculus (pAF), which connects the temporal and parietal cortices (Weiner et al. 2016) (Fig. 1a; for further details on these and other fascicles, see Bullock et al. 2021).

While fascicles by themselves do not have functional properties, they are thought to play an important role in brain function by connecting distant functional regions that are involved in a shared behavior (e.g., reading). Thus, understanding the functional role of fascicles has long-reaching implications for pre-surgical planning, identifying biomarkers of neurological and psychological disorders as well as for understanding brain development, organization, and plasticity, more generally. Ex vivo dissection studies have assigned functional relevancy to fascicles by linking behavioral deficits to lesions of particular fascicles (e.g. Catani and Ffytche 2005; Catani and Mesulam 2008). Additionally, dMRI studies have examined the relationship between behavioral metrics and diffusion metrics of bundles (e.g., the correlation between reading skill and fractional anisotropy in a bundle Yeatman et al. 2011; Vandermosten et al. 2012; Vanderauwera et al. 2017)) to link white matter fascicles to brain function.

In combination, dissection and dMRI approaches have provided valuable, yet somewhat simplistic insights on the functional relevancy of a given fascicle. After all, in both approaches, fascicles are considered as uniform structures that are linked to a specific brain function and/or behavior. The concern with this approach is that it assumes that the entirety of a large anatomical structure such as a fascicle, which is typically several cm wide and can be more than 10 cm in length, is involved in the relevant function. In contrast, functional regions in cortex have a more granular organization. For example, the mosaic of category-selective regions in human ventral temporal cortex (Grill-Spector and Weiner 2014) includes several regions each about ~ 1–2 cm in diameter. As such, the size of each category-selective region in ventral temporal cortex is at a finer spatial scale than the spatial extent of the endpoints of fascicles reaching ventral temporal cortex. This difference is spatial scales is illustrated in Fig. 1b, which shows, in 3 individuals, the relationship between cortical endpoints of the posterior arcuate fasciculus (pAF) and the vertical occipital fasciculus (VOF) in ventral temporal cortex and two visual category-selective regions: face-selective pFus-faces (also called fusiform face area 1) and word-selective pOTS-words (also called visual word form area 1). Critically, endpoints of both the pAF and VOF bundles span a larger spatial expanse than each of the functional regions, and the endpoints of the VOF extend across both face and word-selective regions. This difference in spatial scales suggests that these fascicles likely contribute to multiple functions (e.g., both face and word perception). Thus, we argue that, in order to understand the functional relevancy of fascicles in the brain, it is necessary to directly measure within individuals the set of regions to which each bundle connects.

## White matter bundles are heterogeneous in both structure and functional relevancy

As discussed above, each white matter bundle likely connects regions that span multiple functional networks, suggesting that each bundle may contribute to multiple brain functions. Hence, when researchers analyze an entire bundle as a single entity, they will likely include streamlines that connect to regions from multiple networks. This, in turn, may make the derived measurements, such as the fractional anisotropy, less precise, because researchers may be averaging the measure of interest across streamlines that are involved in different functions and have different structural properties.

**Fig. 2:**
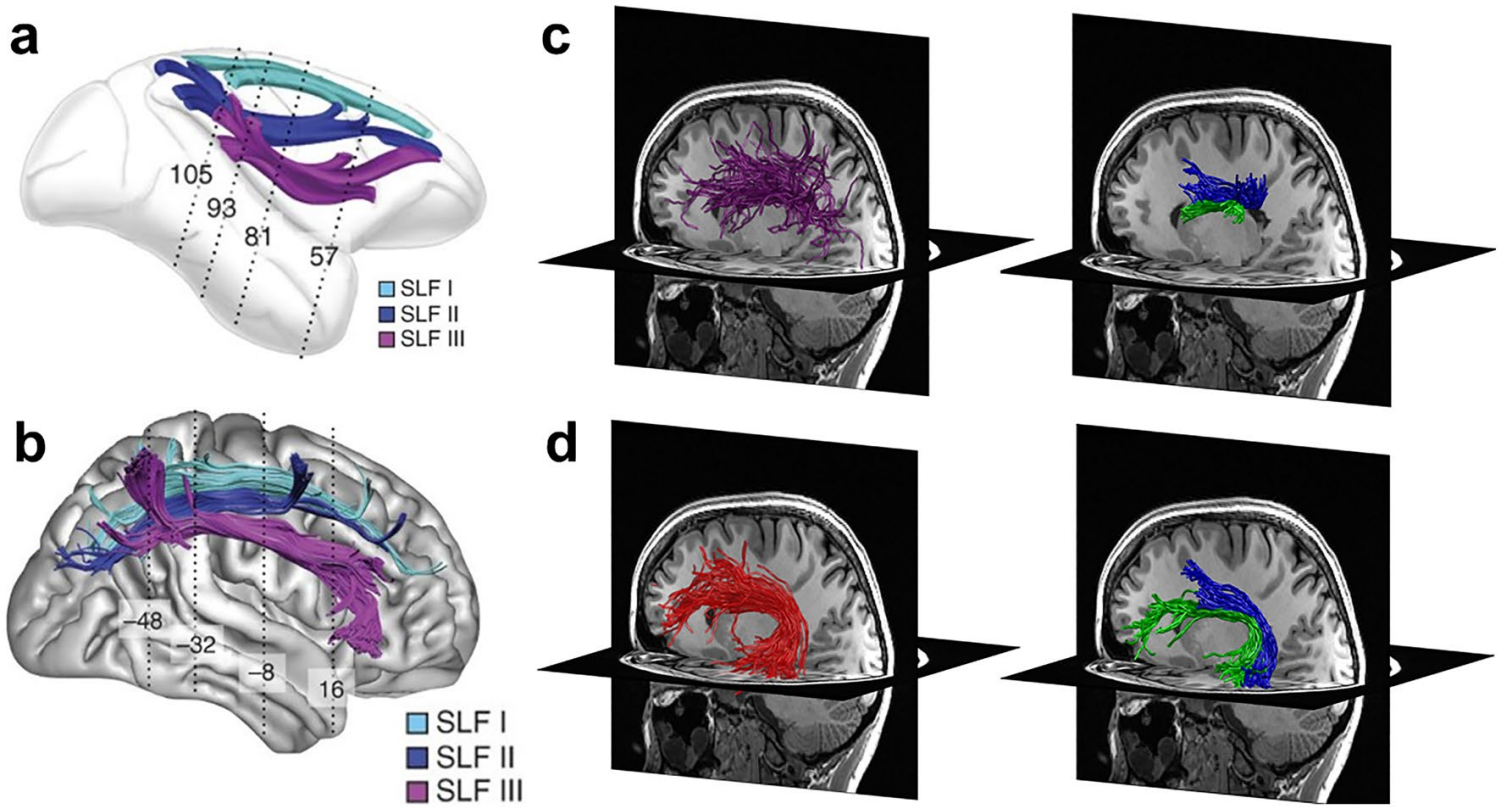
The superior longitudinal fasciculus (SLF) and arcuate fasciculus (AF) contain sub-bundles with distinct structural and functional properties. **a**, **b** Distinct structure: The SLF contains three distinct sub-bundles in monkeys (**a**) and in humans (**b**). Figure reproduced with modification from Thiebaut de Schotten et al. (2011). (**c**–**d**) Distinct function. **c** Comparison of the entire SLF (purple, left column) and sub-bundles within the SLF related to mathematical processing (blue, right column) and reading (green, right column) within the same individual. **d** Comparison of the entire AF (red, left column) and sub-bundles within the AF related to mathematical processing (blue, right column) and reading (green, right column) within the same individual. **c**, **d** Reproduced with modifications from Grotheer et al. (2019). *AF* arcuate fasciculus, *SLF* superior longitudinal fasciculus

The idea that bundles are not uniform in their structural properties, and hence in metrics such as fractional anisotropy, is supported by recent work that has divided large bundles into sub-bundles. These sub-bundles can be identified based on structural criteria, in which case we will refer to them as structurally defined sub-bundles. Bundles may be structurally divided based on different spatial trajectories of groups of streamlines or based on structural differences between groups of streamlines within the bundle. For example, even though it is often considered to be a single bundle, the superior longitudinal fasciculus (SLF) has been divided into 2–3 distinct structural sub-bundles, inspired by findings in non-human primates (Petrides and Pandya 1984; Schmahmann and Pandya 2006) (Fig. 2a). In humans, these structural sub-bundles have been identified based on differences in the spatial trajectories of streamlines (Makris et al. 2005; Thiebaut de Schotten et al. 2011; Wang et al. 2016) (Fig. 2b) or based on differences in the structural properties of streamlines (Schurr et al. 2020). While structurally defined sub-bundles still contain many streamlines, they are likely to be more homogenous than the entire bundle. Interestingly, analyses of the functional relevancy of the structural sub-bundles of the SLF suggest that they show different development trajectories and may be differentially impacted in different cognitive disorders (Galantucci et al. 2011; Thiebaut de Schotten et al. 2011; Parlatini et al. 2017; Fitzgerald et al. 2018; Amemiya et al. 2021). Other examples of structurally defined sub-bundles include: (1) the optic radiation, which contains an anterior sub-bundle that includes Meyer’s loop, which has different structural properties than the rest of the bundle (Schurr et al. 2018), and (2) the anterior limb of the internal capsule, which in both non-human primates and humans contains distinct sub-bundles which vary in their connectivity to different parts of cortex (Safadi et al. 2018).

Sub-bundles can also be identified based on their connections to different functional regions in cortex, which we refer to as functionally defined sub-bundles (fSuB). For example, the SLF contains at least two different fSuB that run roughly in parallel: one sub-bundle consists of streamlines that connect distant cortical regions involved in reading, and the other consists of streamlines that connects regions involved in mathematical processing (Grotheer et al. 2019) (Fig. 2c). Interestingly, these fSuB of the SLF also differ in their structural properties; for example, R1 relaxation rate (which is linearly related to level of myelination Stüber et al. 2014; Kirilina et al. 2020)) is higher in the fSuB of the SLF involved in reading than the one involved in mathematical processing (Grotheer et al. 2019). Similarly, not only the SLF but also the arcuate fasciculus (AF) can be divided into two distinct fSuB (Fig. 2d), one involved in reading and the other in mathematical processing, which also vary in their R1 relaxation rate (Grotheer et al. 2019).

These examples highlight the tight interplay between structure and functional relevancy within individual white matter bundles, as well as the potential benefits of the increased spatial precision gained by dividing large-scale bundles into meaningful sub-bundles. Furthermore, these examples underscore that delineations of large-scale bundles into sub-bundles defined by distinct structural properties, is a promising data-driven approach to gain a finer grained understanding of the organization and functional relevancy of white matter connections (see also Schurr et al. 2018, 2019, 2020). Nonetheless, structurally defined sub-bundles may still connect to regions across multiple functional networks. Hence, we argue that if one’s goal is to understand the functional relevancy of white matter connections, one should directly intersect functional regions and bundles within the same individual, that is directly identify the fSuB of a given functional network.

## Linking functional regions and white matter bundles in the visual system and beyond

Over the past 25 years, researchers have used functional magnetic resonance imaging (fMRI) to identify regions of cortex involved in specific brain functions. Regions associated with a common function are thought to form brain networks, that together support important behaviors like recognizing faces or reading words. While white matter connections themselves are not thought to have functional properties, they nonetheless play an important role in brain function by connecting functional regions of the same network to one another. As such, identifying the structural connections between functional regions of a cortical network is important. After all, structural properties of these connections may impact the efficiency of communication within a brain network, and in turn its function (e.g. Thomas et al. 2009), and structural connections also constrain the location of functional regions (e.g. Saygin et al. 2016).

The human visual system is an excellent model system for examining the link between brain structure and function, as its functional organization is comparatively well understood and easy to probe. As such, during the last 10 years, much progress has been made in understanding the white matter connections of functional regions within the human visual system. This includes studies that explicated: (1) the intricate structural connections between functional regions of the face network (Gschwind et al. 2012; Pyles et al. 2013), (2) different white matter connections associated with face and place processing, respectively (Gomez et al. 2015), (3) the white matter connections of the visual word form area (Yeatman et al. 2013; Bouhali et al. 2014), (4) the white matter connections between early visual cortex and category-selective regions in ventral temporal cortex (Kim et al. 2006), and (5) the white matter connections between early visual cortex and retinotopic visual areas along the IPS (Greenberg et al. 2012).

While earlier studies on the link between functional regions and white matter connections in the visual system predominantly focused on local connections, here, we will focus on a recent expansion of this work that intersects functional regions identified in individual subjects with each individual’s white matter bundles derived from that person’s whole-brain tractogram. We argue that intersecting white matter bundles with functional regions in individual participant’s brains is currently the most precise way to identify which streamlines within a bundle are connected to a specific functional network. It is critical that these analyses are done in individual participant’s native brain space for three reasons (1) it enables higher precision in localizing functional regions of interest (fROIs, e.g. with a fMRI localizer), (2) it preserves relationships between function regions and particular macroanatomical structures like a sulcus or a gyrus, and (3) it preserves inter-individual differences in fROI and bundle size, shape, and location. This sensitivity to inter-individual differences is particularly important when examining special populations (e.g., children or patients) whose functional organization may differ from the general population and hence from available atlases based on brains of typical adults. Further, as this individual subject approach does not require spatial smoothing, which is commonly applied in group analyses but not in individual subject analyses, it further increases the spatial precision of the identified fROIs (e.g., Weiner and Grill-Spector 2013) and the subsequent identification of fSuB. Similarly, while there are stable functional–structural couplings in cortex, whereby functional regions are consistently located in specific sulci/gyri (Van Essen et al. 2012; Weiner et al. 2017, 2018), defining the location of fROIs directly from a functional localizer is more accurate than predicting the region from cortical structure alone. Therefore, there is a limit to the degree to which anatomically defined regions can inform on the link between brain function and white matter connections.

Additionally, focusing on streamlines within bundles, rather than all streamlines in the tractogram, is advantageous as these large-scale bundles have been validated in ex vivo dissection studies. Thus, they are known to exist, which alleviates some of the common concerns regarding false positives in tractography (but see Maier-Hein et al. 2017). Moreover, focusing on these known anatomical structures also facilitates the interpretability of results and comparisons of results across studies. For these reasons, here, we review the promising approach of intersecting fROIs and white matter bundles within individual’s native brain space to identify the functionally defined white matter sub-bundles of the brain.

## How to identify functionally defined white matter sub-bundles?

**Fig. 3:**
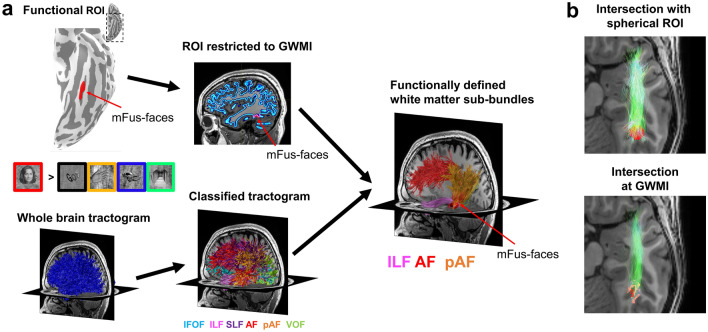
By combining fMRI and dMRI, researchers can identify the functionally defined white matter sub-bundles (fSuB) that connect to a given functional region. **a** An overview of how to identify fSuB within a participant’s brain. First, fMRI data is used to define a functional region of interest (fROI) in the cortex of an individual’s brain. In this example, the fROI is mFus-faces, also referred to as fusiform face area 2. The fROI can then be restricted to those voxels that fall at the gray/white matter interface. Next, dMRI data are used to generate a whole-brain tractogram in the same subject’s native brain space, whereas seeding points for tractography can be placed uniformly across the gray/white matter interface. An example whole-brain tractogram is shown in blue. The tractogram is then classified into known bundles. Finally, by intersecting the fROI with the classified tractogram, the fSuB of that fROI are identified. **b** Comparison of different approaches to intersecting fROIs and classified tractograms. The intersection can be done using spherical ROIs that are centered on the fROI and extend into the white matter (*top*) or directly at the gray/white matter interface underneath the fROI (*bottom*). In this example, mFus-faces (red) is intersected with the inferior longitudinal fasciculus (green) in a representative subject. The fSuB generated at the gray/white matter interface is more precise than that identified using a spherical ROI. *fMRI* functional magnetic resonance imaging, *dMRI* diffusion weighted imaging, *IFOF* inferior frontal-occipital fasciculus, *ILF* inferior longitudinal fasciculus, *SLF* superior longitudinal fasciculus, *AF* arcuate fasciculus, *pAF *posterior arcuate fasciculus, *VOF* vertical occipital fasciculus, *GWMI *gray/white matter interface

To identify fSuB, researchers first use fMRI data to define functional regions of interest (fROI) in an individual’s native brain space (Fig. 3a). For example, they may run a visual localizer (e.g., Kanwisher et al. 1997; Stigliani et al. 2015) to identify category-selective regions in the ventral temporal cortex, such as face-selective regions (fusiform face areas, also called mFus-faces and pFus-faces). Next, within the same individual’s native brain space, a whole-brain white matter tractogram is created using dMRI data. The whole-brain tractogram is then classified into bundles (e.g., using automatic tools Yeatman et al. 2012; Garyfallidis et al. 2018; Grotheer et al., 2021a; Kruper et al. 2021). Next, the classified bundles are “intersected” with the fROI to isolate only those streamlines within each bundle that connect to that fROI, which generates the functionally defined white matter sub-bundles (fSuB) of that fROI. The generated fSuB are hence groups of streamlines from individual bundles that connect to a given functional region.

As outlined above, identifying fSuB requires the intersection of non-overlapping structures within individuals: fROIs that are located on the cortical surface, and white matter streamlines that lie under the cortical surface. As such, identifying fSuB has become feasible only in tandem with substantial improvements of tractography methods (such as constrained spherical deconvolution Tournier et al. 2012) and dMRI data quality (high angular resolution, multi-shell acquisitions, etc.). These methodological advances now enable tracing white matter streamlines all the way to the gray/white matter interface underneath the fROIs, increasing the precision of measurements. Indeed, earlier fSuB studies used spherical ROIs that were centered on fROIs but extended into the white matter to enable intersection with the white matter tractogram (Fig. 3b-top, e.g., Yeatman et al. 2013). As spherical ROIs are shaped differently and are often larger than the original fROIs, they reduce spatial precision. Better spatial precision than spherical ROIs can be achieved using another approach that combines dMRI data with anatomical segmentations of the individual’s brain. In this approach, the subject’s brain anatomy is segmented into gray and white matter and the gray/white matter interface is identified. Next, either the fROI is extended into the white matter or the streamline endpoints are extended into the gray matter, using the gray/white matter interface for guidance (e.g., Lerma-Usabiaga et al. 2018). Even higher precision can be achieved by leveraging anatomically constrained tractography (ACT, Smith et al. 2012). ACT utilizes each individual’s brain segmentation and can seed tractography directly at the gray/white matter interface. This approach allows researchers to intersect streamlines and fROIs directly at the gray/white matter interface without extending either fROIs or streamlines, thereby further improving the precision of measurements (Fig. 3b, Grotheer et al. 2019). Together, these methodological innovations have enabled researchers to link between white matter connections and brain function with unprecedented precision.

When applying the functionally defined sub-bundles approach to study the functional relevancy of white matter connections, researchers should carefully consider several parameters that can impact the identified sub-bundles. These parameters include: the contrast and threshold used for fROI definition, the quality of the dMRI data, and the tractography parameters, as detailed below.

First, when defining functional regions, it is recommended to use a stringent contrast that isolates the specific brain function one wishes to study from related functions. For example, when defining face-selective regions in the fusiform gyrus (pFus- and mFus-faces, also referred to as fusiform face areas), it is important to contrast brain responses to faces with those to many other categories (e.g., places, objects, words, and limbs) as this enables a more precise localization of these fROIs compared to when contrasting faces with only one other category (e.g., objects) (Weiner and Grill-Spector 2010). The statistical threshold used for fROI definition can then be chosen based on the literature to match previous measurements of the average size of the specific fROI. This is important, as larger fROIs will also lead to larger fSuB. Even when using the same contrast and threshold, the size of fROIs will vary between individuals, and it can hence be beneficial to run a control analysis where fROI size is kept constant across participants to ensure that between-subject differences are not related to differences in their fROI sizes.

Second, when classifying tractograms into white matter bundles, researchers should be aware that each generated tractogram depends on the tracking algorithm (e.g., deterministic vs probabilistic) and other tracking parameters (Bastiani et al. 2012; Thomas et al. 2014; Takemura et al. 2016a; Maier-Hein et al. 2017). Furthermore, present tractography algorithms cannot fully resolve crossing-fibers (Roebroeck et al. 2008; Maier-Hein et al. 2017) or accurately project into cortical gray matter (Reveley et al. 2015). To ensure that the identified fSuB reflect the underlying brain connections rather than parameter choices, we recommend generating multiple tractograms with different parameters and testing if and how results vary with the chosen parameters. Additionally, we recommend selecting an approach that identifies bundles which align well with anatomical priors from ex vivo dissection studies. Finally, it is advisable to stay up to date with the ongoing efforts to improve the accuracy of tractography methods (e.g. Smith et al. 2013; Pestilli et al. 2014; Takemura et al. 2016a). After all, as the tractograms become more accurate, so will the identified fSuB.

## What have we learned from functionally defined white matter sub-bundles thus far?

FSuB studies have advanced our understanding of the functional relevancy of the white matter bundles of the brain. One area of substantial recent progress is research on the white matter connections to a reading-related fROI, often referred to as the visual word form area (VWFA, Cohen et al. 2000; Dehaene and Cohen 2011). Importantly, fSuB studies using classified tractograms (Yeatman et al. 2013; Lerma-Usabiaga et al. 2018; Grotheer et al. 2019) revealed a consistent set of bundles (the arcuate fasciculus, vertical occipital fasciculus, inferior longitudinal fasciculus, and posterior arcuate fasciculus) that contain fSuB of the VWFA. These consistent findings across independent researchers, which used different methods to define fROIs, generate tractograms, and intersect the two, provides a striking example of the reliability and robustness of the fSuB approach. Moreover, these studies have generated important conceptual advances by: (1) showing that the VWFA contains two subregions that have distinct fSuB (the anterior VWFA (mOTS-words) connects predominantly to the posterior arcuate fasciculus and the posterior VWFA (pOTS-words) to the vertical occipital fasciculus (Lerma-Usabiaga et al. 2018)), (2) identifying the bundles (the arcuate fasciculus and posterior arcuate fasciculus) that connect the VWFA to other regions of the reading network (Grotheer et al. 2019), and (3) showing that fSuB related to reading and mathematical processing are spatially and structurally segregated within the arcuate fasciculus and the superior longitudinal fasciculus (Grotheer et al. 2019; Fig. 2c,d). Examination of fSuB have also been successfully implemented in non-human primates. For example, in the macaque monkey, this approach has been used to map the fSuB of the attention network (Sani et al. 2019).

Another exciting approach related to fSuB is using white matter bundles within individuals to explicate a functional region’s white matter fingerprint, by grounding it to known white matter structures. The white matter fingerprint approach (Saygin et al. 2012, 2016; Osher et al. 2016) uses the white matter connectivity profile to predict functional maps in cortex. In brief, this method utilizes brain parcellations (e.g., anatomical parcels from FreeSurfer) and measures the amount of pairwise white matter streamlines between each voxel in one parcel to all other parcels in the brain, which is referred to as the white matter fingerprint (Saygin et al. 2012). Then, it finds a linear mapping (weighted sum) between the white matter fingerprint and a functional map of interest. This linear mapping can be used to predict functional maps in any new brain from its white matter fingerprint alone (Saygin et al. 2012, 2016; Osher et al. 2016). While the predictive power of this method is striking, its interpretability is limited, as it does not reveal which white matter bundles constitute the fingerprint that predicts a specific functional map. Thus, an exciting direction is combining the fingerprint approach with the fSuB approach to inform which bundles contribute to the prediction of functional responses. For example, in a recent paper, we combined these approaches and found that a weighted sum of the endpoint densities of the arcuate fasciculus, inferior longitudinal fasciculus and vertical occipital fasciculus successfully predicts distributed fMRI responses in ventral temporal cortex during a reading task, as well as the location of mOTS words (Grotheer et al. 2021b).

Another interesting extension of the fSuB approach is to move from intersecting white matter bundles with functional regions to intersecting them with functional maps. Several studies have combined measurements of retinotopic maps with tractography, providing insights on the retinotopic organization of the white matter (Dougherty et al. 2005; Yoshimine et al. 2018; Kurzawski et al. 2020; Movahedian Attar et al. 2020; Finzi et al. 2021). These studies (1) elucidated the retinotopic organization of streamlines crossing the splenium (Dougherty et al. 2005), (2) mapped short association fibers (u-fibers) in early visual cortex (Movahedian Attar et al. 2020), (3) investigated the effect of macular degeneration on the optic radiation (Yoshimine et al. 2018), (4) evaluated white matter connections between area prostriata and the thalamus, and (5) mapped the connections between face- and place-selective regions and eccentricity maps in early visual cortex (Finzi et al. 2021). While most of these studies focused on local connections rather than bundles, the fSuB approach can easily be adapted to such intersections with continuous functional maps in cortex. This, in turn, may provide an even finer spatial granularity.

Overall, the fSuB approach showed that: (1) the connections of a given functional region to the rest of the brain is typically supported by many white matter bundles rather than a single bundle, (2) a single white matter bundle connects to many functional regions that can be part of distinct functional networks, and (3) combining functional and diffusion MRI data within individuals allows the identification of sub-bundles specific to the brain function of interest, which not only improves precision but also provides a better understanding of the functional relevancy of the brain’s white matter. Despite the progress made to date, the fSuB approach is still in its infancy and there are many additional ways in which it can advance understanding of the functional relevancy of white matter connections. For instance, the improved precision of fSuB may reveal novel biomarkers of neurological and psychiatric disorders that were hidden previously by averaging white matter properties across streamlines that support unrelated brain functions. Additionally, by identifying separate streamlines that are linked to different functions, the fSuB approach may be used to examine how learning and activity-dependent myelination impacts the white matter. Finally, fSuB can be used to study the link between functional development and the development of the white matter. For instance, it can be used to determine if structural differences between fSuB associated with different fROIs are present from birth, or if they develop during the lifetime of an individual.

## Conclusion

We propose that the fSuB approach has the potential to significantly advance our understanding of functional–structural coupling in the visual system and the brain more broadly. As dMRI data quality and tractography approaches improve, so will the anatomical accuracy of the identified sub-bundles, which in turn, will enable researchers to focus on specific streamlines that connect functional regions within the brain network they are investigating. This increased precision will advance understanding of the link between diffusion metrics and behavior in typical as well as clinical populations. The increased precision afforded by the fSuB approach may further facilitate the integration of structural and functional connectivity measures, the detection of biomarkers for different neurological disorders as well as understanding of brain development, organization, and plasticity.
